# A Meta-Analysis of Randomized Controlled Trials on the Effectiveness of Exercise Intervention in Preventing Sports Injuries

**DOI:** 10.7759/cureus.26123

**Published:** 2022-06-20

**Authors:** Okelue E Okobi, Endurance O Evbayekha, Ekene Ilechie, Joy Iroro, Jane N Nwafor, Zinai Gandu, Hameed O Shittu

**Affiliations:** 1 Family Medicine, Lakeside Medical Center, Belle Glade, USA; 2 Internal Medicine, St. Luke's Hospital, St. Louis, USA; 3 Internal Medicine, Calgary Laboratories, Calgary, CAN; 4 Medicine and Surgery, All Saints University School of Medicine, Roseau, DMA; 5 Internal Medicine, University of the District of Columbia (DC), Silver Spring, USA; 6 General Medicine, Ivano-Frankivsk National Medical University, Ivano-Frankivsk, UKR; 7 Internal Medicine, Olabisi Onabanjo University Teaching Hospital, Sagamu, NGA

**Keywords:** eccentric quadriceps work, excercise, exercise training, pediatric sports medicine, sports related injuries

## Abstract

Athletes risk injury every day during practice sessions and actual games, with the majority of the affected population being young males. The Centers for Disease Control and Prevention 2011-2014 report on sport and recreation-related injuries in the United States has consistently shown the average annual estimate of the millions of dollars spent on sport and recreation injuries. These injuries translate to a significant financial implication for the athlete, the team, the health system, and the public health.

We composed a review protocol. We enumerated our inclusion and exclusion criteria, injury definition, and search strategy. We searched PubMed and SPORTDiscus. Then we used Forrest plots for the meta-analysis of the relevant selected studies. We used various keywords in our search strategy. These included “injury,” “sports,” “exercise,” “prevention,” “techniques,” and every possible combination of them. Search results showed 2516 hits with our keywords, and we included 20 of those results.

Twenty trials, including 19712 individuals with 2855 injuries, were analyzed. Eccentric Training relative risk (RR) of 0.54 (95% CI 0.395 to 0.739 with X^2^ of p < 0.05) showed that the risk of the injury was decreased by 54% in the intervention group compared to the control group. In the neuromuscular training group, a RR of 0.682 (95% CI 0.621 to 0.749 with X^2^ of p < 0.001) showed that the risk of the injury was decreased by 68.2% in its intervention group subgroup compared to its control group. Also, the “11” International Federation of Association Football (FIFA) program had a RR of 0.771, indicating that there was a 77.1% decrease in injury by this set of exercises (95% CI 0.728 to 0.816 with X^2^ of p < 0.05), and this “11” FIFA program also had the most preventative effects. Warm-up had a RR of 0.843 (95% CI 0.749 to 0.949 with X^2^ of p < 0.05) and showed small prevention. Strength Training RR of 0.97 (95% CI 0.57 to 1.63 with X^2^ of p > 0.05) had no preventive effect.

Our analysis showed that different exercises have preventive roles in sports injuries. The warm-up FIFA, neuromuscular training, and eccentric training reduced the risk of injury in the intervention group compared to the control group by a high percentage. At the same time, neuromuscular warm-up and FIFA 11 dynamic warm-up also decreased the relative risk of injury in the intervention group. These effects varied among exercise type, injury type, and sport.

## Introduction and background

Athletes risk injury every day during practice, training, and games. According to the Bureau of Labor Statistics, athletes and other people who compete in sports have one of the highest rates of injuries and diseases of any profession [1]. The Centers for Disease Control and Prevention 2011-2014 report on sport and recreation-related injuries in the United States showed an average annual estimate of 8.6 million sport and recreation injuries [2]. The most affected population were males, with 61% of the cases, and persons aged 5-24. Interestingly 3.5 million sports injuries every year happen in a population younger than 18 years old, with children being majorly affected. About one-third of all related childhood injuries are secondary to sports [3].

Participation in sports has a positive impact on public health. Physical activity is vital in preventing and treating most medical conditions. Nevertheless, the incidence of sports-related musculoskeletal injury can muddy this positive effect of sports, especially in youths. These injuries have profound financial implications. For example, sports injuries represent substantial spending for athletes, teams, and the state [4-5]. According to Ryan et al., the financial burden of sports injuries is significant; between 2010 and 2014, hospitalizations for juvenile sports injuries in the United States cost between $10 million and about $44 million [5]; therefore, the prevention of these injuries may be a way to mitigating this cost effect [6]. Sports injury prevention programs might be a way to help tackle this burden. On the flip side, sports injuries are a significant drawback of exercise; however, sports injury prevention programs are accessible and proven in several clinical trials to be beneficial [7].

Different techniques were analyzed in this article to evaluate preventive practices for sports injuries- neuromuscular, eccentric, warm-up, and dynamic training [7]. Some of the studies analyzed in this report focused on one type of intervention or only one kind of sport/physical activity. The relationship between sports injury and preventive exercises has been previously reported. For example, a meta-analysis by Lauersen et al. in 2014 compared the effectiveness of exercise interventions in preventing sports injuries. It included randomized control trials that obtained data on the possibility of combined techniques for prevention [8]. Since then, no other review or meta-analysis has focused on evaluating and comparing all kinds of prevention exercises. New randomized control trials have been published, and more recent techniques and standardized programs have been utilized to prevent injuries since the last review. This review and meta-analysis will broaden the scope of previous studies on sports injuries focusing on different physical activity programs' preventive effects.

Methodology

We composed a review protocol, designed inclusion and exclusion criteria (see Table 1 below), extracted injury definition, and planned a search strategy. All studies were assessed using the domain-based evaluation recommended by the Cochrane collaboration [9]. Study weighting was considered but was not applied; no evidence supports this approach since it would involve subjective decisions [9]. We collected and analyzed various injury types, exercise interventions, and sport type information. We also collected variables necessary to calculate any additional data. A Forrest plot was constructed for meta-analysis of the relevant selected studies. The studies were stratified into less heterogeneous exposure subgroups. We also considered compliance with the study programs [10].

**Table 1:** Inclusion and exclusion criteria

Inclusion criteria	Exclusion criteria
Primary intervention studies were included.	Secondary intervention studies were excluded.
Studies that had no injury at the time of intervention were included.	Studies with baseline pathologies were excluded.
Peer-reviewed randomized control trials were included.	Case reports and review studies were excluded.
Studies that were conducted in humans were included.	Animal studies were excluded.
Studies that had Sports injuries as one of their endpoints.	Other surrogate measures of injury were excluded.
Studies done between 2007 to 2021 were included.	Studies that were done outside 2007 to 2021 were excluded.
Studies that included participants over the age of 12.	Studies done in athletes that were 12 years and below were excluded.
Studies that did not involve the use of equipment during exercise were included.	Studies that used extra techniques (insoles, Kinesio taping, etc.) or extra equipment (bicycles, motorcycles, skies, etc.) were excluded.

The search was conducted on PubMed and SPORTDiscus. The study included randomized controlled trials published between January 2007 and June 2021. Keywords for the inquiry include “injury,” “sports,” “exercise,” “prevention,” “techniques,” and every possible combination of these words. In addition, we customized the searches to accommodate the layout of each search engine.

**Figure 1:**
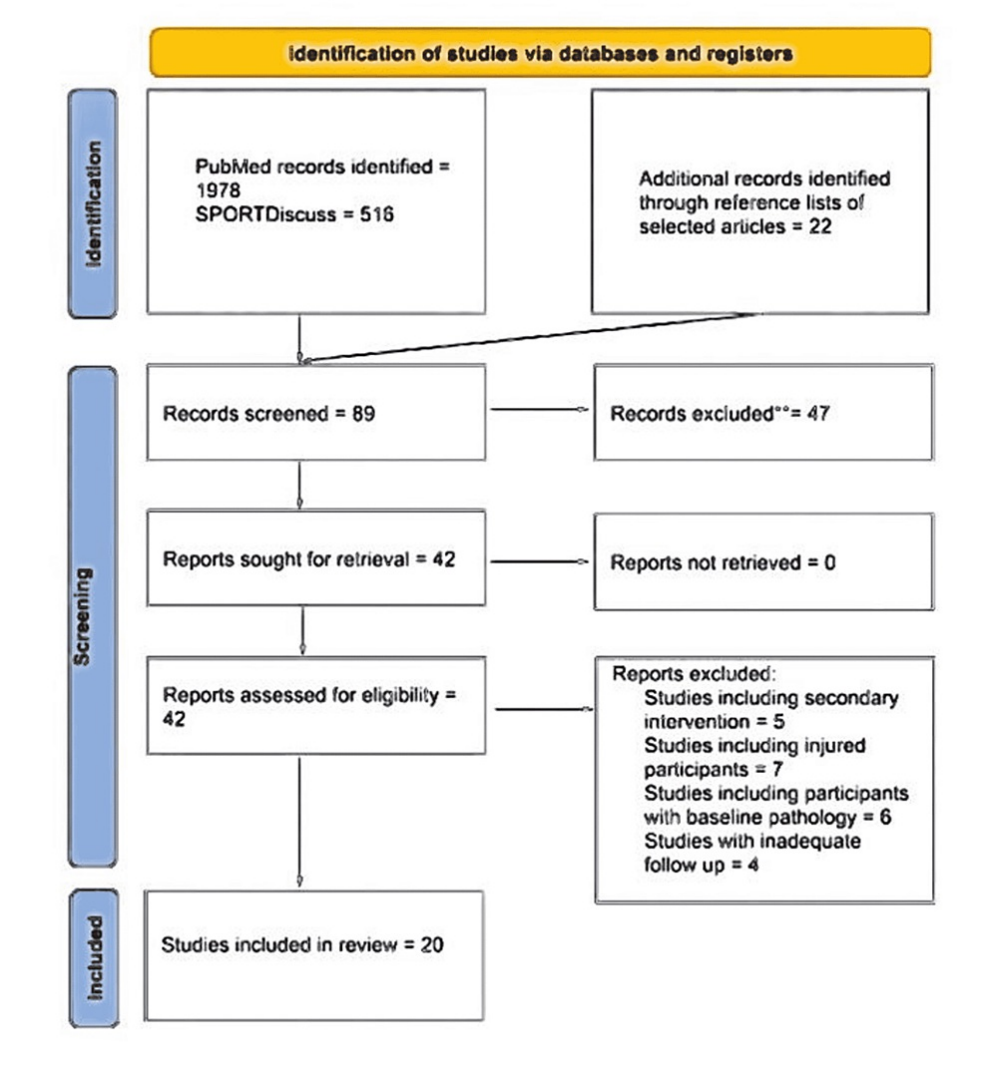
Study Prisma chart

Search results yielded 2516 articles with our keywords, and the results were screened by title having 89 matches. In addition, 20 studies that met the inclusion criteria were reviewed, as shown in the Prisma chart below in Figure 1 below.

The 20 included studies are summarized below in Table 2, which included 19712 individuals in the analysis, and the effects were based on 2855 injuries. The studies evaluated five different kinds of interventions: Eight neuromuscular training, seven utilizing the 11 programs of the International Federation of Association Football (FIFA) (FIFA-11 dynamic warm-up program), two using eccentric training, two for warm-up, and one for strength training.

**Table 2:** Included studies and their characteristics

Studies	Intervention	Population	Completion	Follow-up	Primary Out
Petersen et al. (2011) [11]	Eccentric Training	Danish Male Professional Soccer Players	intervention 461, Control 481	10 Weeks	Hamstring
Van Der Horst et al. (2011) [12]	Eccentric Training	Male Soccer Players	Intervention 292	13 Weeks	Hamstring injury
Emery and Meeuwisse (2010) [13]	Neuromuscular	Male and Female Soccer Players	Control 287, Intervention 364	1 Season	General
Krutsch et al. (2017) [14]	Neuromuscular	Elite Football Soccer Players (Males)	Control 380, Interview 529	14 Months	Severe Knee Injury
Walden et al. (2012) [15]	Neuromuscular	Elite Soccer (Males)	Intervention 2479	7 Months	ACL And Severe
Attwood et al. (2017) [16]	Neuromuscular	Players: Elite Rugby (Males)	Control 2085, Intervention 273	1 Season	Knee Injury Head. Shoulders And
Eils et al. (2012) [17]	Neuromuscular	Players: Basketball	Control 96, Intervention 102	1 Season	Concussion Ankle Injury
Emery et al. (2008) [18]	Neuromuscular	League: Basketball	Intervention 494, Control 426	1 Year	All Injury
Richmond et al. (2016) [19]	Neuromuscular Training	Students in Physical Education	Intervention 361, Control 363	12 Weeks	General
LaBella et al. (2011) [20]	Neuromuscular Warm-Up	Women Soccer And Basketball Team	Intervention 737, Control 755	3 Years	General
Toresdahl et al. (2020) [21]	Strength Training	Marathon	Intervention 273, Control 310	12 Weeks	General
Soligard et al. (2008) [22]	Warm-up	Runners Female Soccer Players	Intervention 1055, Control 837	8 Months	Lower Extremity And Lower Back
Steffen et al. (2012) [23]	Warm-up FIFA 11	Female Soccer Players	Intervention 1091, Control 1001	8 Months	General
Beijsterveldt et al. (2012) [24]	Warm-up FIFA 11	Male Soccer Players	Intervention 223, Control 233	8 Months	General
Hammes et al. (2015) [25]	Warm-up FIFA 11	Male Soccer Veterans	Intervention 146, Control 119	9 Months	General Severe
Longo et al. (2012) [26]	Warm-up FIFA 11	Elite Basketball	Intervention 80, Control 41	9 Months	General
Lopes et al. (2020) [27]	Warm-up FIFA 11	Football; Male Players	Intervention 71, Control 34	20 Weeks	General
Slauterbeck et al. (2019) [28]	Warm-up FIFA 11	Players Athletic Teams High Schools	Intervention 1825, Control 1786	1 Year	General
Silvers-Granelli et al. (2017) [29]	Warm-up FIFA11	Men Soccer Team	Intervention 675, Control 850	14 Weeks: 1 Season	Anterior Cruciate Ligament
Van de Hoef et al. (2019) [30]	Warm-up	Soccer Players First Class Amateur Male	Intervention 229, Control 171	14 Weeks: 1 Season	General

Statistical analysis

Only first-time injuries were considered in all cases of this analysis since repetitive injuries are likely to depend on each other and may introduce bias. We used the RR, Cox regression RR, or injury rate RR to analyze the data. We then adjusted all the studies for clustering effects, five of which the authors did not adjust. The X^2^ p-value assessed the heterogeneity of the analyses. Analyses were computed using Statistical Package for the Social Sciences (SPSS) software (IBM Corp., Armonk, NY).

## Review

Results

**Figure 2:**
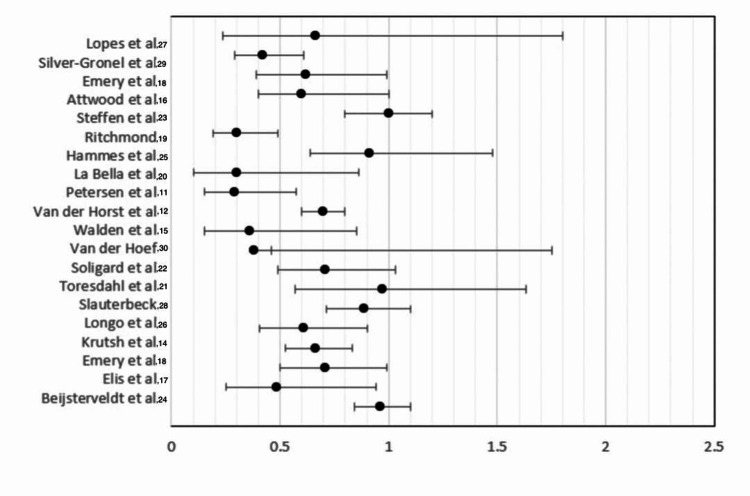
Study Forest plot Petersen et al., 2011 [11]; Van Der Horst et al., 2011 [12]; Emery and Meeuwisse, 2010 [13]; Krutsch et al., 2017 [14]; Walden et al., 2012 [15]; Attwood et al., 2017 [16]; Eils et at., 2012 [17]; Emery et al., 2008 [18]; Richmond et al., 2016 [19]; LaBella et al., 2011 [20]; Toresdahl et al., 2020 [21]; Soligard et al., 2008 [22]; Steffen et al., 2012 [23]; Beijsterveldt et al., 2012 [24]; Hammes et al., 2015 [25]; Longo et al., 2012 [26]; Lopes et al., 2020 [27]; Slauterbeck et al., 2019 [28]; Silvers et al., 2017 [29]; Van de Hoef et al., 2019 [30].

The forest plot is seen in Figure 2 below, and the study characteristic table analyzed 20 randomized control trials that included 19712 individuals with 2855 injuries. The total effect estimate had a RR of 0.672 which means that all the prevention measures had a 67.2% injury reduction rate in the intervention group compared to the control. The 95% confidence interval of RR was 0.43 to 0.870. The data heterogeneity was highly significant, with a chi-square p-value of <0.001. An injury risk reduction of 54% in the intervention group compared to the control group was seen from eccentric training, RR 0.54 (95% CI 0.395 to 0.739 with X^2^ p-value of < 0.05), also, the eccentric training was significantly associated with reduced injuries as the p-value of Chi-square test was < 0.05. In the neuromuscular training studies, the RR of 0.682 (95% CI 0.621 to 0.749 with X^2^ p-value of < 0.001) connotes that the risk of the injury is decreased by 68.2% in the intervention group compared to the control group, which also means that it was significantly associated with reduced injuries as the p-value of Chi-square test was < 0.001. For the “11” FIFA program, the RR of 0.771 indicated that there is a 77.1% risk reduction rate (95% CI 0.728 to 0.816 with X^2 ^p-value of < 0.05). For the warm-up study, a RR of 0.843 (95% CI 0.749 to 0.949 with X2 of p < 0.05) showed significant prevention. Strength training RR of 0.97 (95% CI 0.57 to 1.63 with X2 of p > 0.05) had no prevention effect since the p-value was not statistically significant. These RR values per study are presented in Figure 2.

Looking at the forest plot in Figure 2, most of the studies had RR that conferred protection with a confidence interval (CI) that did not include 1, making the meta-analysis statistically significant. So, although while reviewing the individual studies, they may not have shown any statistical significance in some of the studies analyzed, the clustering through the forest says otherwise.

**Figure 3:**
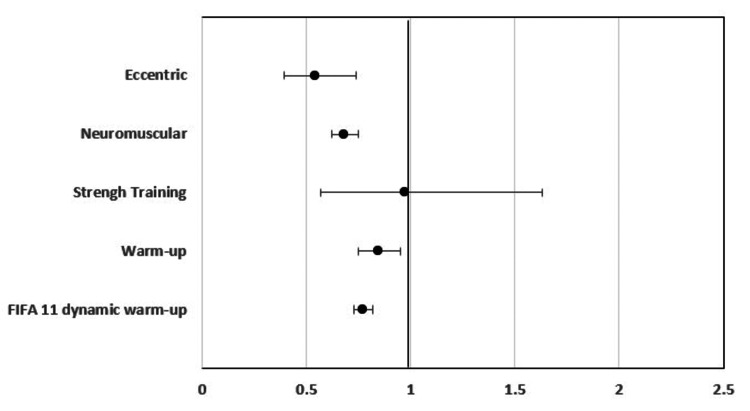
The RR values for each intervention

Different exercises have preventive roles in sports injuries. For example, the warm-up FIFA, neuromuscular training, and eccentric training reduced the risk of injury in the intervention group compared to the control group by a high percentage. At the same time, neuromuscular warm-up and FIFA 11 dynamic warm-up also decreased the relative risk of injury in the intervention group. This effect varies among exercise type, injury type, and sport. The RR values were estimated for each intervention; the results can be seen in Figure 3 below.

The 95% confidence interval (CI) intersected by the vertical line showed that the relative risk (RR) is non-significant for the intervention group compared to the control. The interventions eccentric, neuromuscular, warm-up, and FIFA 11 dynamic warm-up have a significant effect on reducing the risk of injury in the intervention group compared to the control group.

Neuromuscular Training

Eight neuromuscular training studies were evaluated [13-20]: Six of these studies assessed general injuries, while two focused on knee injuries and head injuries, with an estimated RR of 0.682 (95% CI 0.621 to 0.749 with X^2^ of p < 0.001), all studies reported a statistically significant injury risk reduction, but one that [16] reported no apparent effect.

“11” FIFA Program

Seven studies evaluated the dynamic stretch FIFA 11 program [23-29]. Among all the studies being assessed, these were the most homogenous since the length of the intervention and the exercises were standardized in all studies and evaluated general injuries, only varying in the kind of sport: soccer, basketball, and athletic team. An estimated RR of 0.771 (95% CI 0.728 to 0.816 with X^2^ of p < 0.05) was calculated for all studies. Three studies reported no statistically significant results using the FIFA 11 program when evaluating individually, one evaluating an athletic team and two for veteran soccer players.

Warm-up

An estimated RR of 0.843 (95% CI 0.749 to 0.949 with chi-square of p < 0.05) was calculated. Two studies evaluated this category [22,30]. However, the results were the most heterogeneous of all the evaluated exercise groups. One study used males and the other female; both used soccer players and evaluated lower back injuries and general injuries.

Strength Training

Only one study met our inclusion criteria [21]. The value of RR of 0.97 (95% CI 0.57 to 1.63 with X^2^ of p > 0.05) was extracted from the article. No statistically significant difference was observed in the study.

Eccentric Training

Two studies met the inclusion criteria for eccentric training [11-12]. An estimated RR of 0.54 (95% CI 0.395 to 0.739 with X^2^ of p < 0.05). Both studies evaluated comparable populations (male soccer players) and comparable injuries (hamstring injuries). The main differences were the sample sizes, possibly explaining the wide confidence interval.

Discussion

The total effect estimates for physical injury prevention adjusted clustering effects were RR 0.672 (95% CI 0.43 to 0.870 with an X^2^ p < 0.001). In general, the preventative effect of exercise (regardless of its kind) is convincing. The following section discusses the many types of exercise and their preventive effect.

Neuromuscular Exercise

Wide ranges of activities are used in proprioceptive/neuromuscular training to prevent sports injuries. While most studies described neuromuscular training as a combination of balance, weight, plyometrics, agility, and sport-specific exercises performed on stable or unstable platforms with and without postural control perturbations, some authors described it as multi-intervention programs involving a combination [31]. Neuromuscular exercises showed consistent protective effects among all evaluated studies, with an estimated RR of 0.682 (95% CI 0.621 to 0.749 with X^2^ of p < 0.001). Heterogeneity was present, but studies were comparable. Thus, the CI of 95% had a narrow range. This finding aligns with literature reports [31] on neuromuscular exercise for preventing sports injuries; for example, a review [31] evaluated seven randomized clinical trials, demonstrating the effectiveness of neuromuscular/proprioceptive training in reducing the incidence of certain types of sports injuries. Another review [6, 32] evaluated the efficacy of neuromuscular exercise in reducing injuries in soccer players and concluded that neuromuscular training has a protective effect on sports injuries.

"11" FIFA Program

The 11+ program is a complete dynamic warm-up designed to reduce 30-50% of injuries among soccer players aged 14 and older. It is a standardized program that involves injury mitigation emphasized in a workup program called the 11 FIFA, which was developed with the help of the World Football Association’s backing [33]. It consists of 10 exercises emphasizing core stability, dynamic stabilization, eccentric thigh muscle training, proprioceptive training, and plyometrics with straight leg alignment. The bench, hamstrings, chest passing in single-leg stance, cross country skiing, sideways bench, forward bend in single-leg stance, hop over a line, figures-of-eight in single-leg stance, zigzag shuffling, and bounding were among the exercises included in the FIFA 11 program. They are designed to be completed at least twice a week at the start of each training session [33]. In the evaluated studies, the 11+ program had an estimated RR of 0.771 (95% CI 0.728 to 0.816 with X^2^ of p < 0.05), when looking individually, only one study of Slauterbeck et al. reported non-statistically significant findings (including the non-significant RR) [28]. Interestingly, this is the only study of the evaluated group that did not utilize soccer players as recommended by FIFA. In this case, athletic teams were selected. It is impossible to tell from the analyzed studies if the 11 FIFA programs can be extrapolated to other sports types as the studies are too few to make a generalizable conclusion. Additional research is required before a generalizable conclusion can be drawn.

Warm-up

Two studies were evaluated for warm-up [22, 30]. An estimated RR of 0.843 (95% CI 0.749 to 0.949 with X^2^ of p < 0.05) was calculated. This intervention had less protective value out of all evaluated studies, primarily because of the heterogeneity of the trials. Some literature showed that warm-up training has inconclusive evidence of protective value for sports injuries [34].

Strength Training

Lauersen et al. [35] showed the positive benefits of strength training in preventing injuries in their study. Comparative meta-analysis showed that strength training prevention reduced the risk of injury compared to the control group. However, insufficient data satisfied the criteria for drawing clinically meaningful conclusions, as evidenced by the P-value when assessing the study's data from Toresdahl et al. [21]. The value of RR of 0.97 (95% CI 0.57 to 1.63 with X^2^ of p > 0.05) was not statistically significant. Therefore, it is essential to highlight that in this case, participants were educated remotely on how to follow the given strength routine, and no trainers were present to correct or ensure compliance (participants claimed to follow the exercise plan, and compliance was reported as being high). Studies show positive effects of strength training when working on muscles to reduce specific injuries. Perhaps the fact that the evaluated research focused on general injury prevention and not on prevention in the muscle strengthened may have affected the result.

Eccentric Training

Eccentric exercises are a group of workouts demonstrated to develop muscle strength over longer distances by adding sarcomeres in series. These physiological changes protect a muscle from injury by minimizing the damage caused by eccentric contractions. The Nordic hamstring exercise is a widely used and highly effective eccentric activity for hamstring strain prevention. The athlete begins in an upright kneeling position with arms at the sides and a partner supporting the ankles on the ground to do this exercise. The athlete falls forward from this position and, utilizing only the hamstrings, lowers the torso to the ground as slowly as possible. The athlete is prone on the ground, with straight knees, hips, and torso in the finishing position. The sportsman then pushes up with his hands, utilizing only concentric hamstring movements, to return to the starting position. The Nordic hamstring exercise and other techniques can build eccentric hamstring muscle. In general, eccentric training seems to have good results in preventing hamstring injuries. An estimated RR of 0.54 (95% CI 0.395 to 0.739 with X^2^ of p < 0.05) was obtained for our study, reflecting an apparent protective effect. Both evaluated studies utilized Nordic hamstring exercises. Both interventions were homogenous and only varied on sample size. Our findings are in line with the literature results. The eccentric exercise focused on preventing one specific injury instead of general ones. That could be why the exercise's preventive effect is higher in this group.

Study limitations

The main limitation of this analysis was the clinical heterogeneity of the studies selected. Therefore, the results can be clinically useful and interesting observations on the available data. The most effective preventive exercises were neuromuscular and eccentric exercises, with findings consistent with previously available pieces of literature.

## Conclusions

Our research revealed that combining these varied activities could help reduce sports injuries. The warm-up, neuromuscular training, and eccentric training reduced the risk of injury in the intervention group compared to the control group by a high percentage. At the same time, neuromuscular warm-up and FIFA 11 dynamic warm-up also decreased the relative risk of injury in the intervention group. These effects varied among exercise type, injury type, and sport. Future research should compare sports and interventions to determine the best exercise for each sport.
